# Conceptions of human factors in simulation-based education: a phenomenographic study

**DOI:** 10.1186/s41077-026-00462-7

**Published:** 2026-07-08

**Authors:** Alice Kitt, Ranjev Kainth

**Affiliations:** 1 School of Nursing and Midwifery, University of Worcester, Worcester, UK; 2 Faculty of Life Sciences and Medicine, King’s College London, London, UK

**Keywords:** Simulation-based education, Human factors, Phenomenography, Faculty development, Patient safety

## Abstract

**Background:**

Human factors (HF) are integral to patient safety. Simulation-based education (SBE) is widely used to teach HF, yet facilitators conceptualise HF in varied ways. This study explored facilitators’ conceptions of HF.

**Methodology and methods:**

A qualitative phenomenographic study was conducted with 12 SBE facilitators from diverse professional backgrounds. Purposive maximum-variation sampling sought participants from different professions, simulation roles, and educational contexts. Semi-structured interviews were conducted via Microsoft Teams, transcribed verbatim, and analysed iteratively to identify categories of description and an outcome space capturing qualitatively different conceptions.

**Results:**

Four conceptions of HF were identified: (1) HF as a patient-safety science, (2) HF as human-to-human interactions, (3) HF as optimisation of individual performance, and (4) HF as reducing error (‘To Err is Human’). Participants sometimes drew on more than one conception, but usually foregrounded one dominant way of understanding HF. The conceptions differed in scope and alignment with HF theory rather than in simple legitimacy. Dimensions influencing conceptions included professional experience, simulation pedagogy, continuous professional development, and approaches to measuring educational quality.

**Discussion:**

SBE facilitators hold heterogeneous conceptions of HF, risking inconsistent educational practice. In phenomenographic terms, broader conceptions encompassed aspects visible in narrower ones; this should not be read as a claim that one conception is always the ‘best’ for every simulation objective. Rather, different educational aims may legitimately foreground different aspects of HF, while systems-informed conceptions align more fully with contemporary HF science. Clearer definitions, stronger faculty development, and closer collaboration between simulation educators and HF specialists may enhance educational design and patient-safety outcomes.

**Supplementary Information:**

The online version contains supplementary material available at https://doi.org/10.1186/s41077-026-00462-7.

## Introduction / background

Simulation-based education (SBE) has become a central pedagogical approach across healthcare disciplines, enabling learners to develop clinical, cognitive, and interpersonal competencies within safe, authentic environments. Originating from aviation and military training, SBE was adopted by healthcare to enhance technical skill acquisition, team performance, and reduce patient harm [1–5]. Human factors (HF) - a science encompassing psychology, ergonomics, and systems engineering - has increasingly been recognised as an essential underpinning of patient safety and effective clinical performance [6–12]. The two domains share a natural alignment in their emphasis on understanding human performance within complex systems and designing interventions to optimise safety and reliability.

National policy developments in the United Kingdom have firmly positioned both SBE and HF as key enablers of safer healthcare practice. NHS England (formerly Health Education England, HEE) formalised this agenda through the *National Patient Safety Strategy* [13] and *National Patient Safety Syllabus* [14]. These policy documents emphasise the integration of patient safety, SBE, and HF across undergraduate and postgraduate health curricula, as well as in continuing professional development (CPD) for qualified professionals. They mandate that all healthcare professionals acquire and apply HF knowledge as part of their education and clinical practice. The inclusion of SBE within these frameworks highlights its perceived capacity to operationalise HF learning through experiential, reflective, and team-based approaches.

Despite this alignment, how HF is conceptualised and taught within healthcare education remains ambiguous. The term “human factors” is often conflated with “human error” or “non-technical skills” [15, 16]. The breadth of HF’s definition leads some to interpret it as an individual psychological construct focused on behaviour and cognition, while others frame it as a systemic discipline concerned with designing safe environments and resilient organisations [17]. This conceptual inconsistency creates challenges for educators expected to integrate HF principles within their teaching. Without a shared understanding, fragmented or superficial applications may limit the impact of HF education [18].

A growing body of international literature advocates SBE as a mechanism for teaching HF to both qualified clinicians and pre-registration students [3–5, 19, 20]. Empirical studies demonstrate that SBE can improve situational awareness, communication, and teamwork; core domains frequently associated with HF [1]. However, these outcomes often depend on how facilitators design, deliver, and debrief simulation activities. Facilitators’ conceptualisations of HF may influence their learning objectives, fidelity design, and debriefing approaches [21]. Importantly, this need not be framed as a choice between procedural adherence and wider organisational influences. In practice, simulation curricula should be driven by needs assessment: some learning goals may appropriately emphasise procedural reliability or non-technical skills, whereas others require explicit attention to system design, workflow, culture, and organisational learning. The issue, therefore, is not whether one focus excludes another, but whether facilitators recognise the full scope of HF available to them and make deliberate, theoretically coherent choices when designing education.

Much of the existing literature on HF in healthcare education has been descriptive, focusing on skills frameworks or interventions such as Crisis Resource Management or TeamSTEPPS [22, 23]. There is comparatively little research examining how facilitators themselves understand HF and how these understandings shape educational practice. Similarly, research on faculty development within SBE has tended to address technical competence, scenario design, and debriefing rather than conceptual clarity of human factors [24]. This gap in conceptual clarity is notable given that facilitators are the primary mediators between policy intent and learner comprehension.

The application of human factors within healthcare is also relatively recent. Although human-centred design principles were established in aviation and engineering over fifty years ago, their systematic adoption in healthcare education and research has only accelerated since 2010 [10]. Over this period, publications addressing human factors in clinical contexts have increased markedly, signalling both the rapid expansion of the field and its conceptual fragmentation. As scholarship has proliferated, variation in terminology, theoretical scope, and disciplinary interpretation has become more pronounced [25–31]. These divergences likely contribute to the heterogeneity observed in simulation practice and faculty development.

Against this backdrop of expanding research, policy emphasis, and definitional uncertainty, this study explores how facilitators of SBE conceptualise HF. The purpose is to reveal the range of qualitatively different understandings held by educators and how these influence educational design and delivery. This work therefore addresses the following research question:

*How do facilitators of simulation-based education conceptualise human factors?*

By mapping the variation in facilitators’ conceptions, this study aims to contribute to clearer pedagogical alignment between HF and SBE. The findings have implications for faculty-development programmes, curriculum design, and policy implementation, offering insights into how HF knowledge might be more coherently integrated within simulation practice to enhance learning and ultimately patient safety.

### Methodology

Phenomenography was selected to capture the qualitatively different ways in which facilitators experience and conceptualise human factors (HF) within simulation-based education (SBE). Rooted in an interpretivist epistemology, this approach assumes that knowledge is constructed through individuals’ lived experiences rather than measured as objective truth [32–35]. It is particularly suited to exploring variation in perception and meaning, rather than measuring performance or knowledge levels. The method’s outcome; a set of categories of description and an overarching outcome space, presents the collective variation in how a group understands a particular phenomenon [33].

Phenomenography has been extensively applied in higher education to examine teachers’ conceptions of learning, teaching, and disciplinary understanding [32–34]. However, its application within clinical education and simulation research remains limited, offering an opportunity to extend the methodology into an underexplored context. Its strength lies in revealing the underlying qualitative differences in how participants conceptualise a phenomenon, rather than how frequently or accurate those conceptions are [32]. This makes it particularly appropriate for investigating SBE facilitators’ conceptions of HF, which are expected to vary according to professional background, context, and exposure to HF theory and aligns closely with the aim of this study: to illuminate the range and structure of facilitators’ understandings of HF, rather than to evaluate the correctness or completeness of those understandings.

## Methods

### Participants and sampling

Purposive maximum-variation sampling recruited 12 facilitators from diverse healthcare professions, designing, delivering, and debriefing SBE at undergraduate or postgraduate level. Inclusion criteria were current or recent involvement in healthcare SBE facilitation and willingness to discuss HF in relation to practice. In phenomenography, sampling is directed not towards statistical representativeness, but towards identifying sufficient variation in experience, context, and perspective to illuminate the collective outcome space of conceptions.

To support this aim, the sampling strategy intentionally sought breadth across professional background, simulation setting, geographical location, age band, gender, and level of facilitator experience. A broad national recruitment strategy was therefore used to increase heterogeneity in participants’ educational and clinical contexts. This variation is methodologically important because differences in professional role, exposure to systems thinking, organisational responsibilities, and simulation practice may shape the breadth and complexity of awareness through which human factors are understood. By maximising variation in the sample, the study strengthened its ability to identify both narrower and broader conceptions within the phenomenographic outcome space.

An invitation was circulated through the Association for Simulated Practice in Healthcare (ASPiH) members’ email network; the first circulation yielded six responses, two further circulations yielded three additional responses, and a further three participants were recruited through regional simulation networks in London and the West Midlands. Participants were drawn from different institutions and UK regions. Ethical approval was obtained before recruitment from the King’s College London Research Ethics Committee, and all participants provided written informed consent prior to interview. Table 1 summarises the participant descriptors collected for this study.

**Table 1:** Participant descriptors

Participant	Age band	Gender	Clinical background	UK location	Experience in SBE (years)	SBE Context
1	30–40	M	Paramedicine	North East	< 2	Undergraduate
2	30–40	F	Medicine	London/Outer	2–5	Mixed
3	50–60	M	Nursing	South East	2–5	Mixed
4	60+	M	Nursing	South East	5+	Mixed
5	30–40	M	Midwifery	Midlands	< 2	Undergraduate
6	50–60	F	Midwifery	Midlands	< 2	Undergraduate
7	20–30	F	Medicine	London/Outer	5+	Undergraduate
8	30–40	M	Nursing	Midlands	< 2	Undergraduate
9	20–30	M	Nursing	West Midlands	2–5	Postgraduate
10	30–40	M	Allied Health – Operating Department Practitioner	West Midlands	5+	Postgraduate
11	20–30	F	Allied Health - Physiotherapy	West Midlands	5+	Undergraduate
12	50–60	F	Medicine	South West	2–5	Postgraduate

### Data collection

Semi-structured interviews were conducted virtually via Microsoft Teams between March and June 2024, each lasting 45–70 min. An interview guide explored participants’ definitions of HF, examples of how HF was integrated into SBE, views on facilitator preparation, and perceived implications for educational quality and patient safety (Supplementaryfile 2). The guide was developed with supervisory support and refined through a pilot interview with a peer prior to formal data collection; no substantive changes to the topic areas were made thereafter. All interviews were conducted by the first author (AK). At the time of data collection, AK was an experienced simulation educator and Principal Lecturer in Simulation Based Education and Clinical Skills, undertaking postgraduate clinical-education research. AK had no prior personal or professional relationship with the participants. Participants were informed that the interviews were intended to explore different understandings rather than assess knowledge. Interviews were audio-recorded, transcribed verbatim using MS Teams, manually checked for accuracy, and participants were invited to review their transcript for accuracy. Field notes captured contextual details and reflexive observations. The process of sense-checking transcripts supported data familiarisation and formed the first phase of analysis.

### Data analysis

Analysis followed Stenfors-Hayes et al.’s seven-stage framework [35]. Each transcript was read repeatedly for familiarisation, and meaning units were identified, compared, and iteratively grouped to develop categories of description. Coding and primary analysis were undertaken by the first author. Emerging categories, category boundaries, and the developing outcome space were discussed iteratively with the second author and with a peer researcher in clinical education to enhance reflexive challenge and trustworthiness. Where interpretations differed, transcripts and meaning units were revisited until agreement was reached on the most parsimonious description of variation. A summary of the analytic process is presented in Table 2.

**Table 2:** Summary of Data Analysis Process

Step	Stenfors-Hayes et al. [35] description	This research analysis
1 Familiarisation	Reading through interview transcripts to gain a fresh impression; all data initially considered equally.	Interviews conducted via MS Teams; field notes documented; automated transcription verified manually for accuracy and data familiarisation.
2 Condensation	Identifying meaning units within dialogue for further scrutiny; unit size may vary.	Each sentence potentially relevant to meaning was manually coded and highlighted in text.
3 Comparison	Comparing units for similarities and differences.	Notes attached to codes emphasised convergent or divergent views.
4 Grouping	Allocating similar understandings to the same category.	Similar codes grouped under provisional sub-headings. The initial groups were: definitions of human factors; purpose of simulation; influences on facilitation; and perceived outcomes of learning.
5 Articulating	Capturing essential meaning of each category.	Sub-headings merged or refined based on supporting codes; analytic memos documented decisions.
6 Labelling	Expressing the core meaning of each category (steps 3–6 iterative).	Single-word labels assigned to each refined sub-heading to represent essential meaning.
7 Contrasting	Comparing categories contrastively to distinguish boundaries.	Categories examined for counter-examples; peer review confirmed four distinct conceptions.

## Results

Analysis revealed four qualitatively distinct conceptions of human factors (HF) among facilitators of simulation-based education (SBE). These conceptions represent the range of ways in which participants understood the phenomenon, rather than levels of competence, or mutually exclusive positions. Several participants spoke across more than one conception during interview, but usually foregrounded one dominant way of understanding HF.

1. HF as “To Err is Human” – HF equated with individual error prevention, repetition, and vigilance.
2. HF as Performance Optimisation – HF viewed as enhancing cognitive, behavioural, and environmental performance.
3. HF as Human-to-Human Interactions – HF understood as relational and cultural, focusing on teamwork, communication, and civility.
4. HF as Patient-Safety Science – HF conceptualised as a systems-level science integrating ergonomics, design, and organisational learning.

Alongside these categories of description, analysis also identified dimensions of variation [32]. Whereas conceptions describe what HF meant to participants, dimensions capture the key aspects of experience that differed across the conceptions; in other words, the analytic lenses through which the conceptions can be compared and distinguished. Dimensions were derived inductively from the same iterative process of coding and constant comparison: meaning units were compared within and across transcripts, grouped into provisional themes, and then refined to identify the minimal set of cross-cutting aspects that consistently differentiated one conception from another. This process resulted in four dimensions of variation:

- A. Impact of Professional Experience
- B. Pedagogy: Simulation Design and Delivery
- C. Continuous Professional Development (CPD)
- D. Quality: Ensuring and Measuring Effective Education

**Fig. 1:**
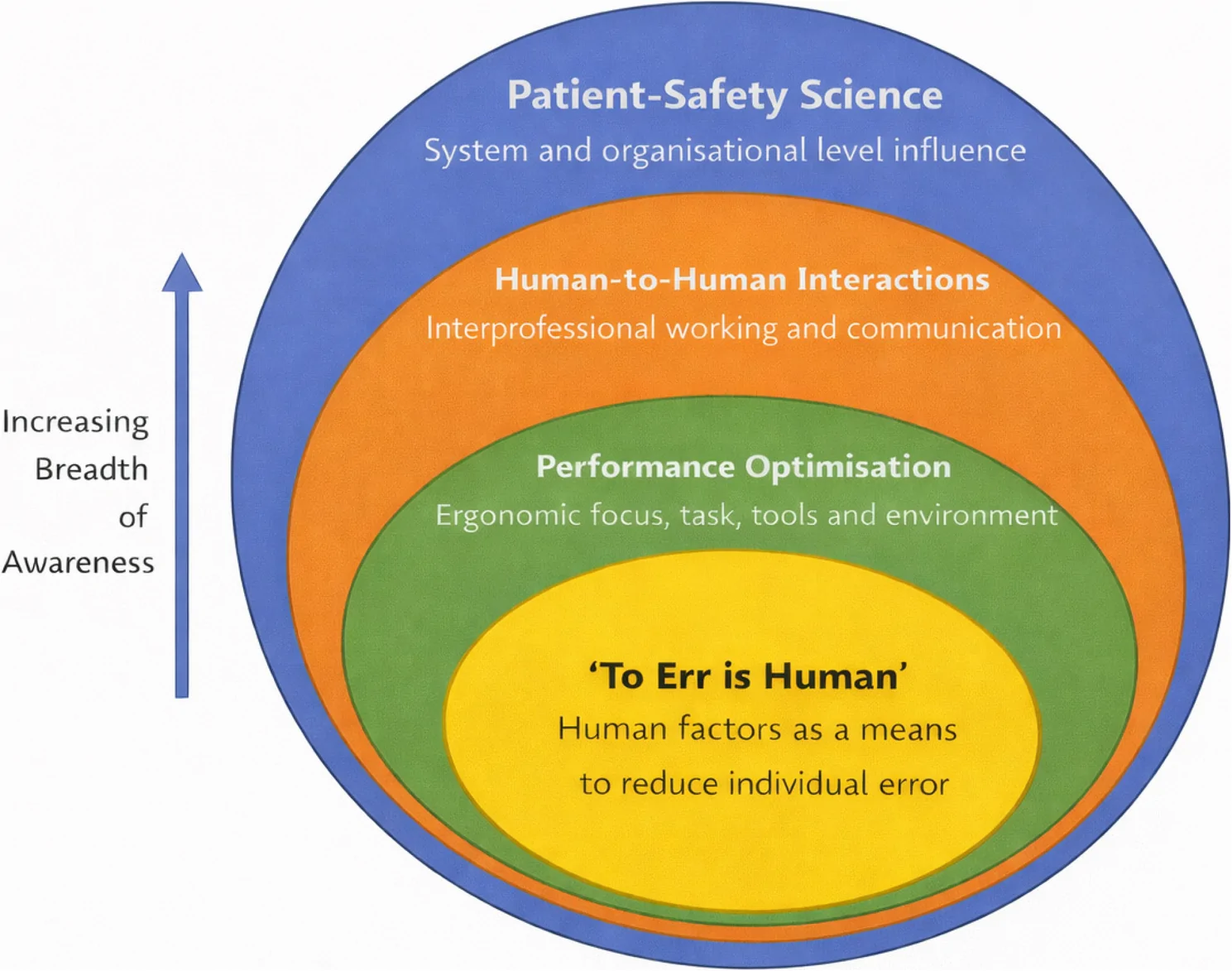
Diagram representing the inclusive and phenomenographic hierarchical relationship between conceptions

The outcome space (Supplementary file 1) represents the structural relationship between conceptions and dimensions. Figure 1 should be read in a phenomenographic sense: the conceptions form an inclusive hierarchy of awareness, in which broader understandings encompass aspects visible in narrower ones. This does not imply that narrower conceptions are without value, nor that every simulation activity should always target the broadest conception. Rather, the figure illustrates increasing breadth of awareness and scope of application in relation to HF theory. Each conception is described through the four dimensions to highlight the distinct, but overlapping, ways in which facilitators conceptualised HF within their practice.

### Dimensions

#### A. impact of professional experience

Participants’ clinical and educational experiences profoundly shaped their understandings of HF. Those aligned with the Patient-Safety Science conception tended to have significant clinical and managerial experience, enabling them to recognise how policy, design, and culture interact to shape safety. They described retrospectively reinterpreting their clinical experiences through a human factors lens once in education.

*“I worked in theatres before the introduction of the WHO checklist and…could directly see the impact on patient safety.”* (Nurse- Postgraduate education).

*“Now I’m in education I see all of it differently… teaching makes me think about how everything was actually related [to human factors].”* (Senior Nurse- Undergraduate education).

This retrospective awareness for educators replicates Schön’s concept of reflection-on-action, where insight arises through later analysis of practice rather than in the moment or “in action” [36].

Those who conceptualised HF as Human-to-Human Interaction**s** were also influenced by their professional experiences but placed greater emphasis on relationships and team dynamics. They regarded teamwork, leadership, and civility as central to patient safety, viewing HF as a lens for understanding how human relationships shape care delivery.

*“Teamworking is the most important factor in patient care… human factors can help us understand what that really means.”* (Midwife- Undergraduate education).

These educators described simulation as a safe environment for examining communication patterns and professional hierarchies, aligning with research linking interpersonal dynamics and civility to safety outcomes [37].

Facilitators expressing the Performance Optimisation conception, often from operational or lone-working contexts such as paramedicine or operating departments, described HF as synonymous with situational awareness and ergonomic efficiency [38].

*“As a paramedic*,* students learn from day one the importance of checking their kit.”* (Paramedic-Undergraduate education).

For them, HF meant preparedness, resourcefulness, and performance under pressure: consistent with ergonomics-based views of HF in healthcare [11]. This conception was narrower in scope, foregrounding optimisation of the individual-in-context more than wider organisational learning.

In contrast, those holding the “To Err is Human” conception tended to view HF as individual responsibility and vigilance. They equated safety with avoiding mistakes and improving technical skill through repetition.

“*People make mistakes all the time and unfortunately those mistakes can mean life or death.*” (Nurse- Undergraduate education).

This conception reflects early, person-based models of error that pre-date systems thinking in patient safety [28–30]. It can support important learning when the immediate aim is safe technical performance, but on its own it captures only a limited portion of the broader HF field.

#### B. pedagogy: simulation design and delivery

Each conception of HF corresponded with a distinctive pedagogical approach to SBE. As suggested in Fig. 1, the pedagogical focus broadened as participants moved from narrower, individualised understandings toward more systems-oriented conceptions. Facilitators expressing the Patient-Safety Science conception displayed deliberate and theory-informed design. They used simulation to provoke reflection on complex systems, integrating both clinical and organisational elements.

“*Simulation has to be purposeful… when human factors is an after-thought*,* it’s far less powerful.”* (Resident Doctor- Postgraduate education).

These educators viewed simulation as part of a broader learning system linking patient experience, safety culture, and service improvement. They advocated collaboration between SBE educators and HF specialists to ensure conceptual accuracy, reflecting the integrative ethos of contemporary safety science [39–42].

Facilitators with the Human-to-Human Interactions conception used simulation primarily for exploring communication, leadership, and teamwork. Scenarios were designed to recreate stressful, interprofessional environments, enabling reflection on behaviours that influence safety.

“*[Simulation] gives learners a chance to practise emergencies*,* explore team dynamics and take that learning back into practice.*” (Consultant Doctor- Postgraduate Education).

Debriefing was viewed as central to learning, with frameworks such as Crisis Resource Management (CRM) used to connect observed behaviours to HF principles [21, 22]. These educators were skilled at drawing relational lessons from practice but tended to stop short of explicit systems-level reflection.

Those conceptualising HF as Performance Optimisation prioritised authenticity of environment and task. Simulation was used to help learners manage workload, equipment, and cognitive stress.

*“We have a simulated ambulance…it shows students how they can work more effectively.”* (Paramedic- Undergraduate Education).

While their scenarios improved individual competence and adaptability, HF was largely framed as personal performance rather than interaction within systems.

Finally, facilitators within the “To Err is Human” conception saw simulation chiefly as repetitive training for error avoidance. Learning was oriented around procedural precision and measurable outcomes.

“The more chances someone gets to practise…the less likely they’ll do harm.” (Operating Department Practitioner [ODP] )

Although effective for technical mastery, this view risked oversimplifying HF as behavioural compliance rather than systems understanding [43, 44]. Importantly, approaches such as deliberate practice and competency-based education remain valuable educational strategies in their own right; the point here is that they should not be conflated automatically with HF science.

#### C. Continuous Professional Development (CPD)

The nature and depth of CPD exposure strongly influenced participants’ conceptualisation of HF. Facilitators with the Patient-Safety Science conception had typically engaged in postgraduate study or contributed to faculty-development initiatives. This exposure developed both conceptual awareness and criticality.

*“My master’s degree incorporated some human factors*,* but it certainly wasn’t a requirement for my role. I now wish I knew more.”* (Senior Nurse- Undergraduate education).

They also expressed concern about the superficial application of HF by those without specialist training.

*“We’re in a somewhat dangerous position where people claim to be experts [in human factors] without proper training.”* (Consultant Doctor- Undergraduate education).

This echoes concerns raised by Russ et al. [16] and Miranda [45], who argue that partial understanding of HF can lead to inappropriate application of its principles in patient safety initiatives.

Those within the Human-to-Human Interaction**s** conception had usually completed shorter SBE-focused training where HF featured as an adjunct topic. They felt confident discussing team-based elements of HF but were less inclined to engage with its scientific or systems-based foundations: their conscious incompetence reminiscent of the Dunning–Kruger model [46].

Facilitators with a Performance Optimisation conception often relied on informal or self-directed learning, inspired by examples from aviation or Formula 1. Their curiosity-driven engagement led to partial and sometimes idiosyncratic understandings of HF principles.

By contrast, those with the “To Err is Human” conception typically had minimal CPD exposure and did not perceive HF as integral to their teaching responsibilities.

“I haven’t had training [on human factors] specifically-it wasn’t required.” (Operating Department Practitioner [ODP]- Postgraduate education)

These findings suggest a developmental gradient in access to, and engagement with, HF ideas: structured, reflective CPD supported more integrated conceptions, while informal or absent CPD potentially reinforced narrower, procedural views [47].

#### D. quality: ensuring and measuring effective education

Participants’ conceptions also shaped how they understood and measured educational quality. Facilitators with the Patient-Safety Science conception defined quality in terms of cultural and system-level impact. They believed education should contribute to a just culture that encourages learning rather than blame [45, 48, 49].

*“Once you see clinical work from a systems-thinking perspective*,* you’re less likely to be reactive and more proactive.”* (Senior Nurse- Postgraduate education).

They linked high-quality education to national standards [50], learner reflection, and patient involvement in simulation design. Quality was thus viewed as a function of organisational learning.

Those with the Human-to-Human Interactions conception equated quality with interprofessional understanding and improved team communication. Success was measured not by technical performance, but by relational growth.

*“If people walk away understanding each other’s roles a little better… then the hospital is already a safer place.”* (Resident Doctor- Postgraduate education).

Facilitators with a Performance Optimisation conception evaluated quality using confidence or competence measures, reflecting an outcome-oriented view of learning. While providing tangible evidence, such measures rarely captured relational or systemic learning outcomes [48].

*“We use pre and post tests to evaluate training.”* (Physiotherapist- Undergraduate Education).

Those aligned with “To Err is Human” equated quality with error reduction and procedural precision. Success was determined by observable performance and the absence of mistakes. This narrow evaluative lens risked reinforcing the belief that safety derives solely from individual compliance rather than adaptive, system-aware practice [30].

## Discussion

This phenomenographic study provides new insight into how facilitators conceptualise HF within simulation-based education. The four conceptions identified - ‘To Err is Human,’ Performance Optimisation, Human-to-Human Interactions, and Patient-Safety Science - represent qualitatively different ways of understanding HF. In keeping with phenomenography, the relationship between these conceptions is hierarchical in the sense of inclusiveness: broader conceptions encompass awareness of aspects present in narrower conceptions. This should not be interpreted as a moral ranking or as evidence that only the broadest conception is educationally legitimate in every context.

### Interpretation of the conceptions

The first conception, ‘To Err is Human,’ mirrors early safety paradigms such as Reason’s Swiss Cheese Model [30], in which human error was regarded as the principal cause of adverse events. Facilitators with this view emphasised repetition, procedural mastery, and technical competence, reflecting traditional behaviourist assumptions about learning [44]. While this approach aligns with competency-based education and skill acquisition theory, it captures HF in a relatively reductive way by centring vigilance and individual performance over the broader systemic and environmental influences on safety [40, 42].

This conception reflects a persistent cultural narrative within healthcare that equates safety with error reduction and individual responsibility. In simulation, this often manifests as skills-based training framed as ‘getting it right for the patient,’ reinforcing the idea that safety derives from competence alone. Such work may be entirely appropriate when the immediate learning objective is technical reliability or early skill acquisition; however, when presented as the totality of HF it risks sustaining blame-oriented assumptions and limiting discussion of system factors.

The second conception, Performance Optimisation, corresponds with ergonomics and cognitive-engineering perspectives that emphasise designing environments and tools to support human performance [27, 40]. Facilitators describing this conception focused on environmental layout, task design, and equipment configuration. Their approach resonates with work on deliberate practice and expertise development [48] and aligns with the human-centred design philosophy embedded in the National Patient Safety Syllabus [14].

This conception demonstrates a shift away from moralising error towards understanding how humans interact with tools and environments. Facilitators adopting this perspective often drew from high-reliability industries such as aviation and motorsport, emphasising efficiency, preparedness, and situational awareness. However, the focus commonly remained at the level of individual optimisation within an existing system, rather than on questioning or redesigning the wider conditions that shape work. Again, this is best understood as a narrower application of HF, not an illegitimate one.

The third conception, Human-to-Human Interactions, reflects sociotechnical and psychological dimensions of HF, highlighting communication, leadership, teamwork, and civility. These facilitators viewed simulation as a social and emotional learning space, consistent with Gaba’s concept of the ‘simulated clinical laboratory’ as a microcosm of healthcare culture [51]. Their emphasis on interpersonal behaviours echoes evidence that communication and relational practice are as critical to safety as technical proficiency [37, 52].

This conception reveals a deeply humanistic understanding of HF, grounded in relationships rather than tasks. Facilitators described simulation as a forum in which professional hierarchies could be examined and empathy, respect, and self-awareness could be practised. While powerful in promoting collaborative learning, this view may underplay material and structural contributors to safety if interpersonal behaviour becomes the only site of explanation. It therefore functions as an important bridge between individual and systems thinking.

The final conception, Patient-Safety Science, represents the broadest and most theoretically integrated understanding identified in this study. Facilitators described HF as an organisational learning process connecting simulation, quality improvement, and safety culture. This systems perspective aligns with contemporary Safety-II approaches [42, 53], which prioritise resilience, adaptability, and learning from everyday work. It also reflects the Chartered Institute of Ergonomics and Human Factors’ advocacy for embedding HF within wider governance and policy frameworks rather than treating it as an isolated educational topic [54].

This conception demonstrates the transformative potential of simulation when educators view it not as a discrete teaching tool but as a mechanism for systemic reflection. Crucially, however, describing Patient-Safety Science as the broadest conception does not mean it is always the only appropriate focus in each simulation. Needs assessment remains central in SBE. Experienced facilitators may quite deliberately foreground technical rehearsal, teamwork, ergonomics, or wider system learning depending on the learning goals; the key issue is whether such choices are made consciously and with conceptual clarity.

### Educational and theoretical implications

The variation identified among facilitators’ conceptions demonstrates that how HF is understood shapes the pedagogy of simulation-based education. Each conception reflects a different scope of awareness and therefore a different educational purpose, design logic, and debriefing emphasis. The findings do not suggest that simulation should always teach HF in the same way; rather, they indicate that facilitators need sufficient conceptual breadth to select and justify an approach that fits the learning context.

Facilitators holding individualistic conceptions of HF - those aligned with ‘To Err is Human’ and, to a lesser extent, Performance Optimisation - tended to design simulation as skill rehearsal. Their scenarios frequently emphasised procedural competence, accuracy, and error prevention. Debriefing in these contexts often focused on technical precision and individual accountability. These sessions can be highly effective for building confidence and procedural fluency, but they may reinforce a performance culture centred on individual mastery rather than collective learning if broader influences remain invisible [17, 20].

Facilitators who conceptualised HF as Performance Optimisation often displayed emerging systems awareness through ergonomic and environmental lenses. Their design priorities centred on realism, equipment use, spatial fidelity, and workload management. This suggests a useful entry point for faculty development: educators already interested in ergonomics can be supported to extend that attention toward workflow, communication pathways, policy, and wider system design.

Facilitators adopting the Human-to-Human Interactions conception engaged with the social and emotional dimensions of HF. Their simulations prioritised teamwork, communication, leadership, and civility, often using interprofessional scenarios to explore how relationships influence clinical decision-making. Faculty development for this group should help connect interpersonal insights to the organisational structures and pressures that shape those interactions.

The Patient-Safety Science conception represents the broadest understanding of HF in relation to contemporary safety science. Educators within this group perceived simulation as part of a wider safety ecosystem linking teaching, service improvement, and organisational learning. Faculty development for this group should focus less on basic definitional clarification and more on sustaining scholarship, collaboration with HF specialists, and links with quality-improvement structures.

Taken collectively, these conceptions can be understood as an inclusive phenomenographic hierarchy, not because one conception has greater worth, but because broader conceptions encompass more aspects of the phenomenon. A facilitator who understands HF as patient-safety science can still choose to focus a session on technical rehearsal or teamwork when appropriate; a facilitator whose understanding remains narrow has fewer conceptual resources available when adapting simulation to different contexts. This distinction is central to the contribution of the paper.

From a curriculum perspective, aligning HF and SBE requires making the implicit explicit. Human factors principles such as workload management, system design, or error recovery, should be consciously designed into scenarios and made visible during debriefings. The *hidden curriculum* of simulation [20] can thus be harnessed to reinforce HF awareness, provided facilitators are equipped to recognise and articulate these dimensions. Interprofessional debriefings, reflective journaling, and learner-led scenario analysis can further embed systems thinking into simulation pedagogy.

At an organisational level, these findings challenge policy assumptions that conceptual coherence already exists among SBE faculty. The National Patient Safety Syllabus [14] presupposes a shared understanding of HF across healthcare education, yet this study shows that conceptions remain fragmented. Aligning educational practice with policy therefore requires conceptual as well as technical development, and may be strengthened by partnerships between educators and HF professionals.

Ultimately, the findings affirm that simulation’s educational impact is inseparable from the conceptual lens through which facilitators view human factors. When HF is understood as a systemic, relational, and reflective science, simulation becomes a transformative mechanism, not merely for individual competence, but for building resilient, learning-oriented healthcare systems [55].

### Limitations

This study has limitations relating to sample diversity, researcher influence, and interview depth. Although participants represented a range of professional backgrounds and UK regions, the sample may not fully reflect the breadth of perspectives across the field. Facilitators with extensive formal HF training or those working in under-resourced settings may have been underrepresented. A further limitation relates to the fact that all interviews and the primary analysis were conducted by the first author. As a solo interviewer-analyst, the researcher’s prior experience, assumptions, and interests in simulation-based education and human factors may have influenced how interview prompts were followed, which meanings were probed in greater depth, and how distinctions between conceptions were interpreted during category development. This creates a recognised risk that the resulting outcome space may partially reflect the researcher’s interpretive lens. Participants may have oriented their responses toward what they perceived to be professionally desirable accounts of HF.

However, within phenomenography, researcher interpretation is not regarded as an undesirable contamination to be eliminated, but as an integral part of making visible the qualitatively different ways a phenomenon is understood. The methodological goal is therefore not researcher neutrality in a positivist sense, but reflexive awareness of how pre-understandings shape the co-construction and interpretation of meaning. In this study, reflexive journaling, field notes, iterative movement between transcripts and emerging categories, pilot interviewing, and supervisory challenge from the second author were used to make this interpretive influence explicit and analytically productive rather than hidden.

### Future research and recommendations

The findings support three immediate implications: the need to develop greater conceptual consensus around HF in SBE, the need for explicit faculty-development opportunities in HF science, and the value of stronger collaboration between simulation educators and HF specialists. Future research should therefore build directly from these contributions by examining how facilitator conceptions influence educational design, learner experience, and organisational learning. Example areas for further study are summarised in Table 3.

**Table 3:** Priority areas for future research arising from the findings

Area	Example research question	Rationale
Facilitator conceptions and learner outcomes	How do differing facilitator conceptions of HF influence what learners understand, retain, and apply in practice?	Would clarify whether conceptual variation translates into differences in educational experience or transfer to practice.
Explicit HF teaching in simulation	What is the comparative impact of implicit versus explicit HF teaching on learner understanding and patient-safety behaviours?	Could inform whether HF should be named and taught directly, or primarily embedded within broader simulation activity.
Collaboration with HF specialists	How does collaboration between simulation educators and HF specialists influence curriculum design, debriefing, and educational coherence?	Could strengthen alignment between SBE practice and HF science.
Faculty-development design	Which faculty-development models most effectively broaden facilitators’ conceptual understanding of HF and support application in SBE?	Would help address current variability in training and support more consistent educational practice.
Shared language and evaluation	What strategies best support shared language around HF in SBE, and what tools can validly assess conceptual understanding and educational impact?	Would support both curriculum development and quality assurance.

## Conclusions

This study illuminates how facilitators of simulation-based education (SBE) conceptualise human factors (HF). The findings demonstrate meaningful variation, ranging from narrow, individual-focused perspectives that equated HF with error prevention to broader, systems-level conceptions aligning more closely with contemporary safety science [12, 18, 25, 42]. In phenomenographic terms, these conceptions form an inclusive hierarchy of awareness.

Facilitators who equated HF primarily with individual error tended to design simulation activities focused on technical competence and error avoidance. While valuable for some learning goals, this approach risks perpetuating outdated notions of blame and personal fallibility if presented as the whole of HF. In contrast, facilitators who embraced broader systems-oriented perspectives designed simulations that prompted learners to consider multiple layers of influence on patient safety, from task design and environmental ergonomics to organisational culture and leadership.

A consistent theme across conceptions was that facilitators’ understanding of HF was often acquired informally, emerging through experience rather than structured education. Those who had undertaken formal training in SBE or HF tended to demonstrate more comprehensive and theoretically grounded pedagogical approaches. This disparity highlights a persistent gap in faculty development: although national strategies strongly promote HF education [13, 14], few structured mechanisms exist to ensure that facilitators receive consistent, evidence-informed preparation.

While simulation is widely endorsed as an effective modality for HF education [1–5, 19], this study suggests that its success depends on the facilitator’s underlying conceptualisation of HF. SBE provides a platform for experiential learning, reflective dialogue, and interprofessional collaboration; however, without conceptual clarity, simulation risks being used only to rehearse procedures rather than also explore systems interactions. Rather than expecting simulation educators to become HF specialists, the findings indicate a need for stronger partnerships between educators and HF professionals [16–20], to ensure pedagogical validity and prevent misinformation.

Ultimately, recognising and addressing conceptual variation among SBE facilitators is essential to achieving coherence between educational intent and patient-safety outcomes. Structured faculty-development initiatives that explicitly engage with HF theory, reflective practice, and systems thinking could strengthen the integration of HF principles across simulation-based education. In doing so, simulation can fulfil its potential not only as a teaching tool but also as a mechanism for cultivating safer, more resilient healthcare systems.

## Supplementary Information

Supplementary Material 1.

Supplementary Material 2.

## Data Availability

No datasets were generated or analysed during the current study.
