# Supplementary material for: Conceptions of human factors in simulation-based education: a phenomenographic study
Source: Adv Simul (Lond). 2026 Jul 8;11:68. doi: 10.1186/s41077-026-00462-7 (PMC13637281; doi:10.1186/s41077-026-00462-7)
Supplement: Supplementary file 1 [file 41077_2026_462_MOESM1_ESM.docx]

**Supplementary file 1- Outcome space**

|  | | Conceptions | | | |
| --- | --- | --- | --- | --- | --- |
| Dimensions | “To Err is Human” | | Performance Optimisation | Human to human interactions | Patient safety science |
| Impact of Professional experience | Humans are flawed, mistakes are inevitable, practice can reduce the incidence of human error    Recognition that clinical work is made up of many individual tasks and skills where errors can occur which can be reduced or mitigated    High consequence of errors in patient care emphasised | | Clinical professionals associated with lone working recognised the importance of tools and   algorithms      Workspace design and physical aspects have a significant role in improving patient care    Simulation gives learners the opportunity to experience clinical tools and resources in a safe setting    Errors can be “designed out” with appropriate equipment | Clinical professionals who worked in complex interprofessional teams recognised the impact of work relationships on patient care for better or for worse    Recognition that team working and communication are significant skills    These skills and human behaviours can be improved with practice    “Civility saves lives” Emphasis on compassion and kindness within high stress environments    Non-technical skills essential in emergency situations  -Decision making, prioritisation, anticipatory thinking | Wider awareness of clinical role as part of complex NHS system    Wider awareness that clinical performance is influenced by many variables    Professionals with greater experience in diverse clinical and/or educational settings    Policies and procedures are implemented to improve patient care across an organisation |
| Pedagogy-  Simulation design and delivery | Simulation is designed to reduce human error through repetition and mastery of skills    Procedural training and feedback      Uni-professional learning    Education referred to as “training” | | Simulation is designed to replicate real environments using real tools | Simulation is designed to replicate real emotions and experiences  -stress creation  -team dynamics    Non-technical skills objectives built into scenarios    Interprofessional education important      Debriefing recognised as most important aspect of learning    Utilisation of specific tools aligned to human factors- Crisis resource management, debriefing models to aid discussion | Critical perspective-SBE is an educational modality which can be useful to share some aspects of patient safety and/or human factors, other modalities can also be effective    Simulations can be adaptive to different and specific learning objectives aligned human factors    Recognition that learning can occur at different phases of a simulation for different learners |
| Continuous professional development | Limited or no formal simulation faculty development or clinical education    Awareness of human factors generated from personal experience and knowledge on human factors not necessarily recognised as important | | Some CPD in clinical education or SBE    Awareness of human factors generated through personal experiences and not education    Knowledge on human factors recognised as potentially beneficial for educational role | Educated in clinical education and/or SBE through some formalised route    Satisfied that they have enough relevant human factors understanding to be effective in their role | Extensive clinical and/or educational experience    Experience developing other educators    Human factors knowledge generated through education    Critical of current education provisions available    Desire for more personal education on human factors and self-aware of potential knowledge limitations |
| Quality- ensuring and measuring effective education | Reduction of error and learners’ ability to perform tasks unaided with full independence    Focus on individual performances    Competency assessments as a performance measure | | Effectiveness measured by learner self-perception of increased confidence as well as competence    Ability to apply skills learned in different settings and scenarios demonstrates understanding, decision making and situational awareness    Using physical equipment and resources effectively | Interprofessional cohesion and mutual respect- understanding each other’s roles    Leadership and followership demonstrable in team performance    Effective communication skills apparent    Dynamic team functioning and responsiveness to stress    Opportunity to practice full complement of non-technical skills | Patient outcome focused, patient voice considered and included from syllabus to delivery    Awareness of national priorities and aligned to standards and policies    Education as a tool to improve culture- culture of learning, a “just” culture    Encouraging systems-level thinking in learners |
