# Supplementary material for: Conceptions of human factors in simulation-based education: a phenomenographic study
Source: Adv Simul (Lond). 2026 Jul 8;11:68. doi: 10.1186/s41077-026-00462-7 (PMC13637281; doi:10.1186/s41077-026-00462-7)
Supplement: Supplementary file 2 [file 41077_2026_462_MOESM2_ESM.docx]

**Supplementary File 2. Interview Guide**

**Interview Schedule**

How do Facilitators of Simulation Based Education conceptualise Human Factors?

**Section 1 – Introduction**

Thank participant for agreeing to participate and attending the interview.

Remind participant of the purpose of the research: to investigate how facilitators of simulation-based education conceptualise human factors.

Confirm that the participant has received the information sheet and relevant national policy documents.

Offer further information and remind participant of confidentiality and anonymity.

Remind participant that the interview is being recorded for transcription purposes only and that the recording will be deleted once transcribed.

**Section 2 – Demographics**

Tell me about your current role within education.

Prompts: position or seniority; regularity of teaching; type of institution; HEI or NHS; type of learner interaction; undergraduate or postgraduate; MDT or uni-professional; management or leadership responsibilities; curriculum design or delivery or both.

How did you discover SBE?

Prompts: career history; experience of simulation as a learner or participant.

**Section 3 – Simulation Based Education**

What do you consider to be simulation-based education?

Prompts: Do you need a sim lab? Actors? Role play vs simulation; clinical skill rehearsal; what is fidelity to you; is it important; mental simulation vs active; use of game theory; use of technology.

What do you think of simulation as a teaching method?

Prompts: benefits and negatives; limitations; when and how does the learning occur; during the prebrief, activity or debrief; role of observation.

Tell me about how you design a simulation session.

Prompts: what considerations do you make; learning outcomes; learning group.

Tell me about how a debrief works in your experience.

Prompts: use a particular model; who leads a debrief; done in pairs or solo?

**Section 4 – Human Factors**

We are now going to talk about Human Factors and Patient Safety in relation to simulation.

Tell me what the term ‘Human Factors’ means to you.

Prompts: human factors vs ergonomics; SHEEP (Systems, Human Interactions, Environment, Equipment, Personal); patient safety; when did you first hear about it; Martin Bromley (CHFG).

Task: We are going to create a list together on these cards, of terms that make up Human Factors. I have written some to get us started. (Use chat box on MS Teams)

Now we are going to put these in a list of most important, to least important.

Explain your ranking.

Is there anything missing from this list?

Do you consider human factors when you design a simulation activity?

Prompts: particular model; CRM; MDT team working; specific focus on ‘non-technical’ skills.

Task: Show National Patient Safety Syllabus (2022) and In Safe Hands Patient Safety toolkit (2019).

When do you think you first saw these documents?

What do you think about them?

Why do you think they came about?

If there are any, what do you think the links are between patient safety and human factors?

**Section 5 – Faculty Development, Quality & Standards**

Tell me about how you ensure high quality in your educational sessions.

Are you aware of any national standards of quality for simulation-based education?

Prompts: Have you heard of ASPiH; membership of any local simulation networks.

Tell me about the training or education you had to use simulation.

Tell me about any education you may have had on human factors.

Prompts: formal or informal.

Have you developed other educators to use simulation-based education?

Prompts: If so, how?

Have you incorporated human factors into educating other educators?

Prompts: If so, how?

How prepared do you feel to talk about human factors with learners?

HEE/NHSE have developed an e-learning package available to all clinical and non-clinical staff on human factors. What do you think about this?

**Section 6 – Other**

What developments do you think will happen next in SBE?

That draws this interview to the end, thank you very much for your time and contribution; do you have any other thoughts or comments before we close?
